# Early Molecular Diagnosis and Comprehensive Treatment of Oral Cancer

**DOI:** 10.3390/cimb47060452

**Published:** 2025-06-12

**Authors:** Po-Chih Hsu, Jen-Hsuan Huang, Chung-Che Tsai, Ya-Hsuan Lin, Chan-Yen Kuo

**Affiliations:** 1 Department of Dentistry, Taipei Tzu Chi Hospital, Buddhist Tzu Chi Medical Foundation, New Taipei City 231, Taiwan; pchsu@gms.tcu.edu.tw; 2 Institute of Oral Medicine and Materials, College of Medicine, Tzu Chi University, Hualien 970, Taiwan; 3 Department of Anesthesiology, Show Chwan Memorial Hospital, Changhua 500, Taiwan; ada064@show.org.tw; 4 Department of Research, Taipei Tzu Chi Hospital, The Buddhist Tzu Chi Medical Foundation, New Taipei City 231, Taiwan; chungche.tsai@gmail.com; 5 Department of Nursing, Cardinal Tien College of Healthcare and Management, New Taipei City 231, Taiwan; 6 Department of Chinese Medicine, Taipei Tzu Chi Hospital, The Buddhist Tzu Chi Medical Foundation, No. 289, Jianguo Rd., Xindian Dist., New Taipei City 231, Taiwan

**Keywords:** oral squamous cell carcinoma, head and neck squamous cell carcinoma, molecular diagnostics, next-generation sequencing

## Abstract

Oral squamous cell carcinoma (OSCC), a major subtype of head and neck squamous cell carcinoma (HNSCC), is a significant global health burden owing to its late-stage diagnosis and poor prognosis. Recent advancements in molecular biology, genomics, and imaging have transformed the landscape of OSCC diagnosis and treatment. This review provides a comprehensive synthesis of early molecular diagnostic strategies, including biomarker discovery using next-generation sequencing, liquid biopsy, and salivary exosomal microRNAs. In addition, we highlight the emerging role of non-invasive optical imaging technologies and their clinical integration for improved surgical precision and early lesion detection. This review also discusses evolving therapeutic approaches, including immunotherapy, neoadjuvant chemotherapy, and patient-centered multimodal regimens tailored through molecular profiling. We emphasized balancing therapeutic efficacy with the quality of life in patients undergoing chemoradiotherapy. The convergence of multi-omics, artificial intelligence, and precision medicine holds promise for revolutionizing early detection and personalized treatment of OSCC, ultimately improving patient survival and clinical outcomes.

## 1. Introduction

Oral cancer, a major subtype of head and neck squamous cell carcinoma (HNSCC), represents a significant and growing global health burden. According to the Global Cancer Observatory (GLOBOCAN), over 377,000 new cases and 177,000 deaths related to oral cavity cancers were reported worldwide in 2020, with particularly high incidence rates in South and Southeast Asia, Eastern Europe, and parts of South America [1]. Histopathologically, oral squamous cell carcinoma (OSCC) accounts for over 90% of oral cancer cases, with common sites of origin including the lateral tongue, floor of the mouth, buccal mucosa, and lower lip [2]. Despite advances in therapeutic modalities, the five-year survival rate for oral cancer remains relatively low, primarily owing to delayed diagnosis and high recurrence rates [3]. Early detection is thus pivotal in improving patient outcomes, as early-stage oral cancers are associated with significantly higher survival rates and reduced treatment-related morbidity [4].

Recent advances in molecular biology and genomic profiling have opened new avenues for early detection and personalized treatment of oral cancer [5]. Biomarkers such as *p53* mutations, *epidermal growth factor receptor* (*EGFR*) overexpression, loss of heterozygosity, alterations in non-coding RNAs, microRNAs, and long non-coding RNAs are being extensively studied as potential diagnostic and prognostic indicators [6,7,8,9,10]. The integration of omics technologies, including genomics, transcriptomics, proteomics, and metabolomics, has significantly enhanced our understanding of oral cancer pathogenesis and heterogeneity [11,12].

Furthermore, liquid biopsy approaches using saliva and blood samples offer a non-invasive strategy for detecting molecular alterations associated with oral cancer [13]. Artificial intelligence (AI)-based tools are increasingly being applied to histopathology, imaging, and molecular data to improve diagnostic accuracy and predict treatment response. These advances align with the paradigm shift toward precision oncology, aiming to tailor therapeutic strategies to the individual genetic and molecular profiles of each patient [14].

While numerous studies have examined the molecular biology and treatment of OSCC, most focus on isolated aspects such as tumor suppressor mutations, individual biomarkers, or specific imaging modalities. In contrast, the present review provides a distinctive and integrative overview that bridges early molecular diagnostics, emerging non-invasive salivary biomarkers (e.g., miR-1307-5p, miR-21), advanced imaging techniques such as structured illumination fluorescence lifetime imaging microscopy (SI-FLIM), and the role of next-generation sequencing (NGS) in tumor stratification and recurrence classification [15,16,17,18,19,20,21,22]. Furthermore, our discussion extends beyond molecular findings to explore the clinical application of patient-centered therapeutic strategies, including oral metronomic chemotherapy, neoadjuvant immunotherapy, and multi-omics-based precision oncology [23,24,25]. This comprehensive approach highlights the practical synergy between diagnostic innovation and therapeutic personalization, setting this review apart from the previous literature and offering novel insights into real-world translational potential.

This review aimed to provide a comprehensive overview of the current and emerging molecular diagnostic tools and therapeutic strategies for oral cancer. We explored the clinical utility of molecular biomarkers, the application of precision medicine in treatment planning, and the potential of immunotherapy, targeted therapy, and traditional approaches for achieving better patient outcomes.

## 2. Molecular Basis of Oral Cancer

### 2.1. Genetic and Epigenetic Alterations

Oral carcinogenesis involves the accumulation of genetic mutations and epigenetic modifications [26]. Key mutations have been reported in *tumor protein p53* (*TP53)*, *cyclin dependent kinase inhibitor 2A* (*CDKN2A)* (*p16INK4A*), and *EGFR* overexpression [27].

#### 2.1.1. Clinical and Molecular Implications of TP53 Mutations in Oral Squamous Cell Carcinoma: Prognostic Significance, Therapeutic Resistance, and Immune Microenvironment Modulation

Clinically, *TP53* mutations are strongly correlated with reduced overall survival and increased resistance to radiotherapy and chemotherapy in patients with HNSCC, highlighting *TP53* mutation status as a potentially valuable biomarker for risk stratification and prediction of therapeutic response in this population [28]. Mutations in *p53* disrupt normal cell cycle control and contribute to oral cancer formation [29]. Detecting *p53* mutations may enable earlier diagnosis and help predict patient responses to radiation and surgical treatment [30]. These findings indicate that truncation mutations in *p53* result in a complete loss of tumor suppressor function, thereby contributing to increased malignancy [31]. Shi et al. reported the role of *TP53* mutations in shaping the tumor immune microenvironment (TIME) in OSCC, a cancer type typically resistant to immune checkpoint inhibitor (ICI) therapy. Using syngeneic OSCC mouse models with defined *Trp53* mutations, researchers found that *p53* mutations led to an immune-excluded cold TIME enriched with immunosuppressive M2 macrophages, resulting in poor ICI responsiveness. Although standard ICI treatment failed, combining programmed death 1 (PD-1) blockade with a stimulator of interferon gene agonist successfully restored antitumor immune responses [32]. A previous study demonstrated a significant association between *TP53* expression and surgical margin status, with higher *TP53* expression observed in cases with positive or close margins. Additionally, *TP53* was universally expressed in cases of lymphovascular invasion (LVI), although the association was not statistically significant, likely because of the limited sample size [33]. Lin et al. reported that most OSCCs exhibit abnormal *p53* expression patterns that are significantly associated with lymph node metastasis at the time of surgery. Using immunohistochemical classification, *p53*-abnormal OSCCs were found to have a markedly higher likelihood of nodal involvement than *p53* wild-type tumors, with a high odds ratio [34]. Summarily, these findings support the use of *TP53* mutation status as a valuable biomarker for early diagnosis, prognostication, and therapeutic decision-making in OSCC, warranting further investigation in larger prospective clinical studies (Table 1).

#### 2.1.2. Implications of *CDKN2A* Alterations in Oral and Head and Neck Squamous Cell Carcinoma: Distinguishing Somatic Events from Hereditary Risk

Based on these findings, *CDKN2A* mutations do not appear to significantly contribute to inherited susceptibility in patients with multiple primary squamous cell cancers of the head and neck. Although loss of heterozygosity at the *CDKN2A* locus is common in sporadic HNSCC, the absence of coding mutations in this high-risk cohort suggests that germline *CDKN2A* alterations are unlikely to be major predisposing factors. Therefore, routine germline *CDKN2A* testing is not warranted for hereditary risk assessment in this patient population [35]. The inactivation of p16^INK4a^, a tumor suppressor protein encoded by the *CDKN2A* gene, has been widely implicated in the development of OSCC [36]. This study highlights a potential link between germline *CDKN2A* c.301G>T mutations and increased susceptibility to OSCC, contributing to the growing evidence that *CDKN2A* mutation carriers may be at an elevated risk for HNSCC. These findings support the consideration of *CDKN2A* genetic testing in families with a history of HNSCC or young patients lacking traditional risk factors. Furthermore, routine surveillance for HNSCC should be implemented in individuals with known *CDKN2A* germline mutations to enable early detection and improve clinical outcomes [37]. A longitudinal study revealed that homozygous deletion of exon 1α of the *CDKN2A* gene, which encodes the tumor suppressor p16^INK4a^, is a frequent event in oral carcinomas and can also occur in a subset of precancerous lesions. In contrast, deletions involving exons 2 and 1β, which affect both p16 and p14^ARF^, are less common. Notably, the patterns of allelic imbalance vary between precancerous lesions and their corresponding carcinomas, suggesting that oral carcinogenesis may follow a genetically heterogeneous and nonlinear progression pathway [38]. Therefore, although routine germline *CDKN2A* testing may not be universally indicated, it should be considered in familial or early-onset cases, and surveillance strategies should be emphasized for individuals with known germline mutations to improve early detection and clinical outcomes (Table 2).

#### 2.1.3. *EGFR* Overexpression in Oral Cancer: Prognostic Significance, Genetic Variants, and Emerging Therapeutic Strategies

*EGFR* overexpression is associated with poor prognosis in oral cancer, contributing to a malignant phenotype characterized by inhibited apoptosis and enhanced metastatic potential [39]. Chen et al. demonstrated that *EGFR* overexpression is significantly associated with advanced tumor stage, nodal involvement, extracapsular spread, and poorer survival outcomes in patients with OSCC, particularly in a population with a high prevalence of betel quid chewing, smoking, and alcohol use [40]. A systematic review and meta-analysis demonstrated that *EGFR* overexpression was significantly associated with poorer overall survival, increased likelihood of lymph node metastasis, and poor tumor differentiation in patients with OSCC [41]. A retrospective study by Laimer et al. confirmed that *EGFR* overexpression was a significant independent prognostic marker in patients with oral and oropharyngeal squamous cell carcinoma. High *EGFR* expression, observed in over 70% of the tumor samples, was significantly associated with reduced overall survival, and multivariate analysis identified it as an independent risk factor [42]. It has been implied that the *EGFR* intron-1 CA repeat polymorphism alone is not directly associated with *EGFR* overexpression and that the presence of short CA repeats in both alleles (SS genotype) notably increases OSCC risk, particularly in areca quid chewers. Furthermore, EGFR protein overexpression was independently correlated with aggressive tumor features, including poor differentiation and nodal metastasis with extracapsular spread. Critically, patients with both *EGFR* disomy and the SS genotype coupled with *EGFR* overexpression exhibited the poorest survival outcomes, suggesting a synergistic effect [43]. However, this editorial explored the limitations and future directions of *EGFR*-targeted therapies for HNSCC. Although *EGFR* is overexpressed in over 90% of HNSCC cases, traditional treatments, such as cetuximab (a monoclonal antibody) and tyrosine kinase inhibitors (TKIs), have shown limited clinical efficacy, largely due to low *EGFR* mutation rates, acquired resistance, and the activation of alternative oncogenic pathways. A novel combination therapy using osimertinib, a third-generation EGFR-TKI, and dihydroartemisinin (DHA), a repurposed antimalarial drug, demonstrated synergistic antitumor effects in preclinical models by suppressing key resistance mechanisms, such as STAT3, Src, and AXL signaling [44]. Overall, these findings highlight the need for biomarker-guided therapies and innovative combination strategies to improve outcomes in patients with *EGFR*-overexpressing OSCC and HNSCC (Table 3).

#### 2.1.4. The Emerging Role of MicroRNAs as Diagnostic, Prognostic, and Therapeutic Biomarkers in Oral Squamous Cell Carcinoma

MicroRNAs (miRNAs) are small non-coding RNAs that regulate gene expression and play key roles in tumor initiation, progression, and metastasis by modulating critical pathways, including the epithelial–mesenchymal transition (EMT). Abnormal miRNA expression has been observed in oral cancer, and certain miRNAs are promising diagnostic and therapeutic biomarkers [9]. The expression patterns of specific miRNAs are positively correlated with clinical stage, metastasis, and patient survival, suggesting their potential as prognostic biomarkers of oral cancer [45]. Both upregulated (onco-miRNAs) and downregulated miRNAs contribute to tumorigenesis by targeting genes involved in cell proliferation, apoptosis, and drug resistance. In silico and functional studies support their biological relevance. miRNA expression signatures have shown promise in diagnosis, staging, prognosis, and predicting response to chemotherapy, particularly to agents like cisplatin and doxorubicin. Circulating miRNAs also have potential as non-invasive biomarkers [46]. Using qRT-PCR analysis, Rajan et al. reported that upregulation of miR-21, miR-196a, and miR-1237 and downregulation of miR-144 and miR-204 were significantly associated with poor prognosis. The miR-196a/miR-204 ratio was the strongest predictor of disease recurrence and patient survival [47]. Recently, salivary miRNAs have emerged as a promising avenue for the early detection, prognosis, and treatment of oral cancer [15]. Similar results were obtained using data from The Cancer Genome Atlas (TCGA) and small RNA sequencing, followed by qRT-PCR validation in saliva samples, and the miRNA panel showed high diagnostic accuracy [16]. Another study utilized public Gene Expression Omnibus datasets to analyze miRNA and gene expression profiles in oral cancer (OC), identifying 23 differentially expressed miRNAs (DEmiRs) and 1233 differentially expressed genes (DEGs). Functional and pathway analyses revealed that these DEmiRs and their targets were heavily involved in cell cycle regulation, DNA replication, and several oncogenic signaling pathways, including insulin-like growth factor, platelet-derived growth factor, liver kinase, and polo-like kinase 1. Protein–protein interaction (PPI) network analysis highlighted key hub genes that are directly linked to cell cycle control and suggested a mechanistic role for miRNAs in OC tumor progression [48]. A systematic review revealed the prognostic potential of miR-21, miR-155, and miR-375 as biosignatures in HNSC. However, TCGA data validated that the differences in the expression levels of these miRNAs showed a modest association with overall survival, indicating potential prognostic relevance [49]. Jakob et al. were the first to report that hsa-miR-99b-3p and hsa-mirR-100-5p are promising prognostic biomarkers for OSCC in a European population. Their differential expression patterns correlated with distinct clinical outcomes and may serve as molecular tools for patient stratification and personalized therapy [50]. Similar results have shown that circulating miR-31-5p may function as an independent diagnostic biomarker for oral cancer and hold potential as a therapeutic target [51]. Yu et al. demonstrated a significant association between miR-21 overexpression, PTEN dysregulation, and perineural invasion (PNI) in OSCC. High miR-21 levels were found to be an independent predictor of poor disease-free survival, suggesting its role as a marker of aggressive tumor behavior [52]. The study evaluated the expression of salivary miR-21, miR-155, and miR-375 as prognostic and predictive biomarkers in 61 patients with stage II–IV OSCC. miR-21 and miR-155 levels were significantly elevated, whereas miR-375 levels were reduced in patients with OSCC compared to those in healthy individuals. The expression levels of these miRNAs were correlated with tumor size (T index) and lymph node metastasis. Notably, patients who responded well to neoadjuvant chemotherapy (NACT) had lower miR-21 and miR-155 levels and higher miR-375 levels in their saliva [53]. Summarily, these findings highlight the significant promise of miRNA profiling as a powerful tool for enhancing early detection, forecasting treatment responses, and personalizing therapeutic strategies in patients with OSCC (Table 4).

## 3. Early Molecular Diagnostic Approaches

The standard diagnostic workflow for OSCC typically involves clinical examination followed by histopathological confirmation via scalpel biopsy. Adjunctive tools such as toluidine blue staining, autofluorescence visualization, and brush cytology are sometimes used to guide biopsy sites or detect potentially malignant disorders [28,29]. However, these techniques suffer from limited sensitivity and specificity, particularly in identifying early or subclinical lesions [30]. Moreover, the invasive nature of biopsy procedures often delays diagnosis in asymptomatic individuals and contributes to patient non-compliance. These diagnostic challenges are further compounded by tumor heterogeneity and the lack of reliable prognostic indicators, which hamper early risk stratification and personalized intervention [6,14]. Consequently, there is a critical need for non-invasive, cost-effective, and highly sensitive diagnostic tools that can detect OSCC at its earliest stages, distinguish aggressive subtypes, and monitor recurrence. Advances in salivary diagnostics—including exosomal miRNAs such as miR-1307-5p—and genomic technologies like NGS offer promising avenues for overcoming these limitations and enabling precision screening and early detection [15,20,54].

### 3.1. Salivary and Blood-Based Extracellular Vesicles, Exosomal miRNAs, and Circulating Tumor DNA (ctDNA) as Emerging Non-Invasive Biomarkers for Early Detection, Prognosis, and Therapeutic Targeting in OSCC and HNSCC

Non-invasive detection of ctDNA, exosomal microRNAs, and tumor-derived extracellular vesicles (EVs) in saliva and blood shows promise for the early detection and monitoring of OSCC and HNSCCs [49,55,56,57]. Salivary biomarkers, including IL-8, Cyfra21-1, and MMP-9 have high sensitivity and specificity [58,59]. This scoping review evaluated salivary molecules as potential biomarkers for the early detection, prognosis, and malignant transformation of OSCC. The most frequently studied and promising candidates included TNF-α, IL-1β, IL-6, IL-8, LDH, and MMP-9, all of which demonstrated high sensitivity and specificity in identifying OSCC and oral potentially malignant disorders (OPMDs) [60]. The study investigated the expression of five salivary mRNA biomarkers, including *IL-1β*, *IL-8*, *SAT*, *S100P*, and *OAZ1*, using qRT-PCR to evaluate their potential in detecting early-stage OSCC and distinguishing it from oral potentially malignant disorders (OSF and OLP) and healthy controls. The combined biomarker panel achieved a 100% predictive probability for OSCC, demonstrating strong diagnostic accuracy [61]. Hu et al. explored the utility of cell-free RNA (cfRNA) transcriptome profiling from saliva and blood as a non-invasive diagnostic approach for OSCC. While no OSCC-specific RNAs were detected in the blood samples, upregulated CLEC2B and downregulated DAZL, F9, AC008735.2 were detected and showed significant differential expression in patients with OSCC compared to those with benign tumors and healthy controls. Additionally, immune profiling has revealed altered immune cell infiltration patterns in the saliva of patients with OSCC [62]. Therefore, ctDNA offers a promising, non-invasive approach for the early diagnosis, prognosis, and monitoring of HNSCC, particularly when used alongside emerging biomarkers, such as exosomes and methylation profiles [63] (Table 5).

In this study, we identified miR-1307-5p as a potential salivary exosomal biomarker for oral cancer using small RNA and transcriptome sequencing and validated it using TCGA data and qRT-PCR. miR-1307-5p is significantly overexpressed in both oral cancer tissues and salivary exosomes and is associated with poor survival, disease aggressiveness, progression, and chemoresistance. Bioinformatics analysis revealed that miR-1307-5p likely contributes to cancer progression by downregulating tumor-suppressive genes, such as *THOP1*, *EHF*, *RNF4*, *GET4*, and *RNF114* [17]. In contrast, exosomal miRNAs have emerged as promising biomarkers for HNSCC detection. Among these, miR-10b-5p, miR-486-5p, miR-24-3p, miR-412-3p, and miR-512-3p show strong potential for diagnostic applications, whereas miR-1307-5p and miR-519c-3p are notably overexpressed and associated with poor survival outcomes, highlighting their prognostic relevance [64]. Similar results showed that microarray analysis identified 109 differentially expressed miRNAs, with miR-24-3p significantly upregulated in patients with OSCC. Elevated expression was confirmed by qRT-PCR and was associated with strong diagnostic accuracy. Functional studies have shown that miR-24-3p promotes OSCC cell proliferation by regulating cell cycle-related genes and directly targeting Period 1 (PER1) [18]. A systematic review assessed the potential of salivary miRNAs as non-invasive biomarkers for detecting early malignant transformation in OPMDs and OSCC. The findings highlighted several dysregulated salivary miRNAs, including miR-21, miR-27b, miR-24, miR-31, and miR-184, that were upregulated, whereas miR-145, miR-200a, miR-191, and others were downregulated. Notably, miR-184 showed the highest diagnostic accuracy, whereas miR-21 and miR-145 were significant in distinguishing OPMD from OSCC and healthy tissue [65]. Patel et al. identified salivary exosomal miR-1307-5p as a promising biomarker for oral cancer using small RNA and transcriptome sequencing, which was validated using TCGA data and real-time PCR. miR-1307-5p is significantly overexpressed in oral cancer tissues and salivary exosomes and correlates with poor survival, disease progression, tumor aggressiveness, and chemoresistance. Bioinformatics analysis suggested that miR-1307-5p promotes cancer progression by suppressing key tumor suppressor genes such as *THOP1*, *EHF*, *RNF4*, *GET4*, and *RNF114* [54]. Therefore, salivary exosomal miRNAs, particularly miR-1307-5p, have strong potential as non-invasive biomarkers for early detection, prognosis, and monitoring of oral cancer progression. Their integration into clinical practice could significantly enhance personalized treatment strategies; however, larger, standardized clinical studies are necessary to fully validate their diagnostic and prognostic utility (Table 6).

This pilot study evaluated salivary exosomes as non-invasive biomarkers for the early detection of OSCC. Exosomes were isolated from saliva samples of healthy controls, patients with OPMD, and patients with OSCC. Whole-mouth saliva collection yielded the highest concentration and quality of exosomes. Mass spectrometry analysis identified specific salivary exosomal proteins, including PSB7, AMER3, and LOXL2, that could distinguish patients with OSCC from healthy controls with high accuracy. Biological pathway analysis suggested that these proteins are involved in tumor progression [19]. Recent studies have indicated the role of EVs in the diagnosis, progression, and treatment of OSCC, a highly aggressive cancer with a poor prognosis. The traditional diagnostic and treatment methods for OSCC are invasive and often ineffective. EVs, which participate in intercellular communication and reflect the tumor status, offer a less invasive alternative for early diagnosis and monitoring. Additionally, this study explored how EV-based strategies, such as inhibiting EV internalization and engineering therapeutic vesicles, may provide novel therapeutic approaches [67,68]. This study evaluated salivary EV-associated miRNAs as potential non-invasive biomarkers for OSCC. Using a qRT-PCR array and validation in additional cohorts, four miRNAs, miR-302b-3p, miR-517b-3p, miR-512-3p, and miR-412-3p, were found to be either uniquely expressed or significantly upregulated in patients with OSCC compared to controls [66]. Li et al. explored the role of EVs derived from OSCC cells in tumor progression. OSCC-derived EVs were shown to promote tumor growth by altering the TME, increasing levels of inflammatory cytokines, including IL-17A, IL-10, IL-1β, and immune checkpoint molecules, including programmed death-ligand 1 (PD-L1), and activating the IL-17A signaling pathway, including TRAF6 and c-FOS. In contrast, inhibiting EV release with GW4869 suppressed tumor growth, enhanced liquefactive necrosis, reduced inflammatory cytokines, and shifted the tumor towards a less invasive phenotype. OSCC-derived EVs also modulate CD8^+^ T cell responses and immune suppression through PTPN2 regulation [69]. Overall, salivary EVs and exosomes hold significant promise as diagnostic, prognostic, and therapeutic targets for OSCC. They offer a less invasive alternative to traditional biopsy-based methods and play an active role in tumor biology (Table 7).

### 3.2. Advances in Next-Generation Sequencing for Genetic Profiling, Diagnosis, and Prognosis in OSCC and HNSCC

NGS offers powerful insights into the genetic and molecular landscape of OSCC, which could greatly improve early diagnosis, prognosis, and treatment strategies [70]. One review emphasized the underutilization of NGS in the study of OSCC, despite its potential to uncover critical genetic alterations and miRNA dysregulation. This article highlights commonly identified mutations in *TP53*, neurogenic locus notch homolog protein 1 (*NOTCH1)*, and *phosphatidylinositol-4,5-bisphosphate 3-kinase catalytic subunit alpha* (*PIK3CA)* and discusses the role of specific miRNAs, such as miR-21, in OSCC progression. It also explores emerging approaches, such as targeted gene therapy and the potential of miRNAs as diagnostic and therapeutic biomarkers [71]. Dongre et al. developed and validated a custom-targeted NGS panel to detect frequent mutations in archival Formalin-Fixed Paraffin-Embedded (FFPE) tissue samples from patients with HNSCC. Despite varying tissue preservation times, mutation detection remains reliable. Frequent mutations were identified in *TP53*, *FAT1*, and *FLG* in HPV-negative tumors and in *FLG*, *FAT1*, and *FGFR3* in HPV-positive tumors. The presence of cancer-specific mutations was correlated with poor differentiation, aggressive invasion patterns, and shorter survival outcomes, as corroborated by TCGA data [20]. One study analyzed mutations in the first and second primary tumors (SPTs) from 13 Taiwanese patients with oral cancer using NGS. By comparing genetic profiles, researchers have differentiated between true second primary tumors and cancer recurrences. Unique driver gene mutations have been identified in several cases, leading to the reclassification of some clinically diagnosed recurrent cancers as new primary cancers and vice versa. The key mutated genes identified were *SYNE1*, *TP53*, and *CDKN2A*. Molecular diagnosis based on driver and trunk mutations has proven helpful in accurately distinguishing tumor types, for which traditional clinical and pathological methods are often misclassified [21]. Therefore, NGS provides critical insights into the molecular drivers of OSCC and HNSCC and offers powerful tools for early diagnosis, prognosis prediction, treatment personalization, and better tumor classification. Broader adoption of NGS in clinical and research settings can significantly enhance the precision and outcomes of oral cancer management (Table 8).

### 3.3. Advancements in Optical Imaging for Early Detection, Surgical Guidance, and Diagnosis Optimization in HNSCC and OSCC

Optical imaging technologies are being developed to overcome the limitations of current diagnostic and treatment methods for HNSCC [72]. Traditional biopsy and surgery face challenges owing to subtle early mucosal changes and difficulties in accurately determining surgical margins. Optical imaging techniques, such as fluorescence imaging, narrow-band imaging, Raman spectroscopy, optical coherence tomography, hyperspectral imaging, and photoacoustic imaging, offer promising non-invasive solutions for the early detection, intraoperative guidance, and therapeutic monitoring of HNSCC [73]. Fluorescence molecular imaging is more accurate than narrowband imaging (NBI) for intraoperative tumor margin delineation in OSCC surgery, especially for detecting submucosal extension. NBI is a practical and cost-effective tool for the treatment of early-stage tumors. Both imaging methods improve surgical outcomes compared with standard care and can reduce tumor-positive margin rates, thus enhancing patient prognosis [74]. One study explored the use of SI-FLIM to improve the early detection of OSCC. SI-FLIM enhances traditional FLIM by reducing out-of-focus fluorescence, allowing for more accurate depth-resolved imaging of metabolic changes that are specifically characterized by NADH fluorescence in the oral mucosa. Using a hamster model, SI-FLIM demonstrated a superior ability to differentiate mild dysplasia, which was identified as an early precancerous change from normal tissue, compared to wide-field FLIM, with a higher diagnostic accuracy [22]. Conversely, Romano et al. discussed the critical need for earlier and more accurate diagnosis of OSCC, given its high mortality and the limitations of conventional biopsy methods. Non-invasive imaging techniques, including vital staining (toluidine blue or Lugol’s iodine), tissue autofluorescence, NBI, optical coherence tomography, high-frequency ultrasound, and in vivo confocal microscopy have been evaluated for their ability to improve early detection, diagnostic precision, and patient compliance. Each method offers specific advantages, such as real-time tissue characterization and visualization of vascular patterns. The authors proposed a three-step diagnostic protocol that integrates these non-invasive methods before proceeding to biopsy, aiming to speed up diagnosis, reduce unnecessary biopsies, and improve surgical planning [75]. Hence, non-invasive optical imaging technologies have significant potential to improve the early detection, diagnostic precision, and intraoperative management of HNSCC and OSCC. Integrating these tools into the clinical workflow, particularly through multimodal strategies, can enhance surgical accuracy, reduce the need for invasive procedures, and ultimately lead to better patient outcomes (Table 9).

## 4. Integrative Treatment Strategies and Patient-Centered Management in Oral Squamous Cell Carcinoma

Surgical resection should be adjusted based on the tumor stage and individual characteristics. Narrower margins may be appropriate for early-stage tumors, whereas wider margins might be necessary for advanced cases owing to greater microscopic spread [76]. Concurrent chemoradiotherapy (CCRT) has been associated with improved survival outcomes in patients with pT2N1 oral cancer compared with postoperative radiotherapy (PORT). In contrast, patients with pT1 disease did not experience significant survival benefits from CCRT over PORT [77]. CCRT with cisplatin and docetaxel appears to be a safe and effective treatment for advanced oral cancer, offering outcomes comparable to those of standard CCRT for head and neck cancers with a potentially lower incidence of severe toxicity [23]. In contrast, a cohort study demonstrated that refusing adjuvant therapy (AT) after surgical resection of advanced OSCC is significantly associated with worse oncological outcomes [78]. Adjuvant radiotherapy is used in advanced stages or for patients with positive margins or nodal involvement [79].

Chemotherapy plays an important role in the multimodal treatment of OSCC, particularly in advanced-stage disease, recurrent tumors, or cases unsuitable for surgical resection [80]. The primary goals of NACT are to reduce tumor size, facilitate more conservative surgical procedures, enhance overall survival outcomes, and potentially improve patient quality of life (QOL) [81]. Additionally, neoadjuvant chemotherapy in locally advanced OSCC may reduce margin positivity rates, thereby improving the likelihood of achieving clear surgical margins. However, this approach has not demonstrated significant benefits in terms of overall survival or response rates [82]. Neoadjuvant immunotherapy, especially with ICIs such as pembrolizumab and nivolumab, has shown promise in reducing tumor size and converting previously unresectable cases into operable ones [24]. Recurrent or metastatic head and neck squamous cell carcinoma (R/M HNSCC) presents a major clinical challenge with limited improvements in survival in recent decades [83]. Oral metronomic chemotherapy (OMCT), typically using low-dose methotrexate and celecoxib, is a low-toxicity, cost-effective, and non-intravenous alternative for patients who are ineligible for or awaiting standard chemotherapy or surgery. Its mechanisms involve antiangiogenic effects, immune modulation, and tumor growth suppression, making it suitable for both palliative and potential adjuvant/neoadjuvant settings [84]. Management must be individualized, considering factors such as PD-L1 status, performance status, toxicity profile, and patient QOL, especially in platinum-refractory cases [83]. Recent efforts have explored the role of immunotherapy and personalized chemotherapy guided by molecular profiling using multi-omics technologies in HNSCC [25]. Patients with oral cancer often encounter significant challenges following CRT, which can be broadly categorized into physiological and psychological effects. Physiologically, CRT frequently leads to mucositis, pain, nutritional deficiencies, and a decline in QOL. It also contributes to eating difficulties, including nausea, vomiting, dysphagia, and impaired digestion, all of which further compromise a patient’s well-being. CRT is associated with an increased risk of depression, anxiety, and emotional distress. Collectively, these complications profoundly affect the overall QOL of patients undergoing oral cancer [85]. Thus, future therapeutic strategies must balance efficacy with tolerability, emphasizing patient-centered care and supportive interventions to optimize clinical outcomes and overall well-being (Table 10).

## 5. Discussion

Despite advances in diagnostic tools and therapeutic options, early detection and comprehensive management of OSCC remain challenging. First, early-stage OSCC often presents nonspecific or asymptomatic lesions that are difficult to distinguish from benign oral conditions, resulting in delayed diagnosis [86,87]. Visual inspection alone lacks sufficient sensitivity and specificity, and there is a lack of widely adopted non-invasive biomarkers for early detection. Secondly, the heterogeneous molecular and immune landscape of OSCC complicates treatment decisions, particularly for patients with lymph node metastases or perineural invasion [88]. Furthermore, current therapeutic strategies such as surgery, radiotherapy, and chemotherapy can lead to significant functional and aesthetic deficits, impacting the patient’s quality of life [89]. Lastly, there is an unmet need for integrated, personalized approaches that combine molecular diagnostics, targeted therapy, immunotherapy, and supportive care to improve survival and long-term outcomes [90,91].

## 6. Summary

This review highlights recent advances in early molecular diagnosis and comprehensive treatment of OSCC, a prevalent and aggressive subset of HNSCC. The integration of molecular biomarkers, including genetic mutations and non-coding RNAs, with liquid biopsy techniques and multi-omics platforms, including genomics, proteomics, and metabolomics, has opened new avenues for early detection, personalized treatment, and prognosis prediction of OSCC. Non-invasive diagnostic tools such as salivary exosomes, ctDNA, and EVs have shown high sensitivity and specificity, offering practical alternatives to traditional biopsies.

Advances in NGS have provided detailed insights into tumor heterogeneity and molecular subtypes, enabling precision oncology. Moreover, optical imaging technologies, such as fluorescence molecular imaging, narrow-band imaging, and structured illumination FLIM, enhance surgical guidance and intraoperative margin assessment. Neoadjuvant chemotherapy and immunotherapy are emerging therapeutic options, particularly for locally advanced or unresectable cases. OMCT offers a low-toxicity regimen for patients who are ineligible for conventional treatment. Importantly, this review also addresses the significant physiological and psychological effects of treatment, particularly chemoradiotherapy, on patients’ QOL, advocating for individualized and supportive care strategies.

## 7. Conclusions

Incorporating early molecular diagnostics and personalized multimodal treatment strategies is promising for improving the outcomes of OSCC. Although substantial progress has been made in biomarker discovery, imaging technologies, and systemic therapies, future clinical implementation will require standardized validation studies, integration of emerging technologies, and a patient-centered approach to optimize therapeutic efficacy and QOL. Despite significant advances in understanding the molecular pathogenesis of oral cancer, several challenges persist. These include the lack of standardized, clinically validated biomarkers for early detection, difficulties in integrating multi-omics data into routine practice, and limited therapeutic options for advanced-stage and treatment-resistant cases. Recent evidence has identified Paraoxonase-2 (PON2) as a novel and promising biomarker in OSCC. PON2, a mitochondrial and endoplasmic reticulum-associated enzyme with antioxidant properties, has been reported to be significantly overexpressed in OSCC tissues compared to normal oral mucosa [92]. This overexpression is associated with enhanced cell survival and proliferation, likely through the modulation of oxidative stress and mitochondrial function [93]. Notably, PON2 has been implicated in mediating resistance to both cisplatin and radiotherapy by mitigating therapy-induced oxidative damage and apoptosis [94]. These findings suggest that PON2 not only holds diagnostic potential but may also serve as a therapeutic target to overcome treatment resistance in OSCC. Future research focusing on the clinical utility of PON2 inhibition could offer new avenues for improving treatment outcomes in resistant OSCC cases.

Despite notable advances in understanding the molecular landscape of OSCC, significant challenges remain. These include the heterogeneity of tumor biology, limited clinical validation of molecular biomarkers, and barriers to implementing real-time, non-invasive diagnostics in routine care [18,19,21]. The integration of next-generation sequencing, salivary exosomal miRNAs, and advanced imaging modalities such as SI-FLIM shows promise but requires further standardization and longitudinal clinical trials [16,17,22]. Additionally, patient access to precision oncology tools and individualized treatment protocols remain uneven across healthcare systems.

Future directions should focus on validating multi-analyte salivary biomarkers in large, multicenter cohorts; enhancing point-of-care platforms for early detection; and embedding artificial intelligence in diagnostic interpretation and clinical decision-making [20,23]. Furthermore, translational research must bridge laboratory discoveries with real-world applications, ensuring that novel diagnostic and therapeutic approaches such as oral metronomic chemotherapy and neoadjuvant immunotherapy are safe, accessible, and effective across diverse patient populations [24,25]. A multidisciplinary and patient-centered strategy that unites molecular diagnostics, digital technology, and evidence-based therapeutics will be essential for transforming oral cancer care in the coming decade.

Additionally, inter-patient heterogeneity and TME complexity continue to impede the development of universally effective interventions. Moving forward, future research should prioritize the validation of salivary and circulating biomarkers in large, multicenter cohorts; refinement of non-invasive diagnostic tools, such as liquid biopsy; and the integration of AI-driven algorithms for predictive modeling. Equally important is the advancement of targeted and immunotherapies tailored to specific molecular subtypes of oral cancer. A multidisciplinary approach combining molecular science, digital health innovation, and clinical oncology will be essential to transform early detection and to personalize treatment strategies, ultimately improving survival and quality of life for patients with oral cancer.

Looking forward, the personalization of OSCC treatment remains a critical but incompletely realized goal. Despite increasing knowledge of the tumor’s genomic and immunologic heterogeneity, translation into individualized therapy is still limited. Key unresolved questions include how to integrate multi-omics biomarkers (genomics, transcriptomics, immunophenotyping) to accurately stratify patients for targeted therapies or immunotherapies; how to overcome resistance mechanisms in high-risk or recurrent OSCC; and how to balance therapeutic efficacy with preservation of oral function and quality of life. Moreover, there is a need for robust predictive models and prospective trials that incorporate molecular profiles to guide treatment selection [95,96]. The development of precision medicine approaches such as patient-derived organoids, tumor-infiltrating lymphocyte profiling, and real-time monitoring through liquid biopsy may help optimize clinical outcomes and minimize overtreatment [97,98].

## Figures and Tables

**Table 1:** Clinical and molecular implications of *TP53* mutations in OSCC.

Key Findings	Clinical Implications	References
*TP53* mutations lead to treatment resistance and poor survival	May guide personalized treatment and prognosis	[28,29,30,31,32,33,34]
Immune evasion via cold tumor microenvironment (TME)	Poor ICI response	[32]
Associated with lymphovascular invasion and positive surgical margins	Biomarker for risk stratification	[33]

**Table 2:** Role of *CDKN2A* alterations in OSCC and HNSCC.

Key Findings	Clinical Implications	References
Germline mutations rare in sporadic cases	Routine testing not recommended broadly	[35]
Germline variants in young or familial cases	May justify targeted surveillance	[37]
Deletion of exon 1α linked to OSCC	Early genetic marker for progression	[38]

**Table 3:** *EGFR* overexpression in oral cancer.

Key Findings	Clinical Implications	References
Overexpression correlates with nodal spread and poor survival	Prognostic marker	[39,40,41,42]
*EGFR* CA repeat polymorphism (SS genotype) increases risk	Risk stratification in specific populations	[43]
Limited efficacy of current *EGFR*-targeted therapies	Need for novel drug combinations	[44]

**Table 4:** Diagnostic and prognostic potential of miRNAs in OSCC.

miRNAs	Expression Pattern	Clinical Application	References
miR-31	Strong predictor of recurrence	Prognostic signature	[15,16]
miR-21	Prognostic markers in European cohort	Patient stratification	[16,47,48]
miR-125a-5p	Associated with tumor size and nodal status	Prognostic and predictive biomarkers	[48]
miR-145	Linked to chemoresistance and poor prognosis	Salivary exosomal biomarker	[48]
miR-223	Upregulated	Involved in cell cycle regulation	[48]
miR-155	Upregulated	Immune regulation; involved in tumor progression; reported in multiple studies	[48,53]
miR-196a, miR-1237	Upregulated	Strong predictor of recurrence; prognostic signature	[47]
miR-1444, miR-204	Downregulated	Strong predictor of recurrence; prognostic signature	[47]
miR-99b-3p, miR-100-5p	— (not specified)	Patient stratification in European cohort	[50]
miR-375	Upregulated	Correlated with tumor size and nodal status; prognostic and predictive marker	[53]
miR-1307-5p	Upregulated	Associated with chemoresistance and poor prognosis; salivary exosomal biomarker	[54]
Panel (e.g., miR-31, miR-21, miR-133a)	Mixed patterns (up/down)	Early diagnosis of OC with high specificity and sensitivity	[15,16]

**Table 5:** Salivary and blood biomarkers for early detection of OSCC.

Biomarker Type	Specific Markers	Clinical Utility	References
Cytokines and proteins	IL-8, MMP-9, CYFRA21-1	Early detection with high sensitivity	[58,59,60]
mRNA panel	*IL-1β, IL-8, SAT, S100P, OAZ1*	100% predictive accuracy for OSCC	[61]
cfRNA (saliva)	CLEC2B ↑, DAZL, F9, AC008735.2 ↓	Non-invasive diagnostic tool	[62]

↑: upregulation; ↓: downregulation.

**Table 6:** Salivary exosomal miRNAs in OSCC diagnosis and prognosis.

miRNA	Expression	Clinical Significance	References
miR-1307-5p ↑	Poor prognosis, chemoresistance	Prognostic biomarker	[17]
miR-24-3p ↑	Promotes proliferation via PER1 targeting	Diagnostic potential	[18]
miR-302b-3p, miR-517b-3p	Unique to OSCC patients	Diagnostic biomarkers	[66]
miR-412-3p, and miR-512-3p ↑	Discriminative power in ROC analysis	Screening tools	[66]

↑: upregulation; ↓: downregulation.

**Table 7:** Salivary EVs and exosomes in the diagnosis and treatment of OSCC.

Study/Authors	Focus	Key Findings	Clinical Significance	References
Pilot study on salivary exosomes	Early diagnosis	Exosomal proteins PSB7, AMER3, and LOXL2 identified in whole mouth saliva (WMS) samples; high diagnostic accuracy	Non-invasive detection of OSCC using salivary biomarkers	[19]
EV-based approaches in OSCC	Diagnosis, monitoring, therapy	EVs participate in tumor communication; reflect disease status	Less invasive alternative to biopsy; potential for real-time monitoring	[67,68]
miRNA profiling in salivary EVs	Diagnostic biomarkers	miR-302b-3p and miR-517b-3p uniquely expressed; miR-512-3p and miR-412-3p significantly upregulated	Promising non-invasive miRNA biomarkers for OSCC screening	[66]
Li et al.	Tumor progression mechanisms	OSCC-derived EVs increase IL-17A, IL-10, IL-1β, PD-L1; activate TRAF6 and c-FOS; inhibition via GW4869 reduces malignancy	EV modulation may serve as a therapeutic target; immune reprogramming potential	[69]

**Table 8:** Applications of NGS in oral and head and neck squamous cell carcinoma.

Study/Authors	Focus	Key Findings	Clinical Implications	References
General review on NGS in OSCC	Early diagnosis and therapy	Underutilization of NGS in OSCC; recurrent mutations in *TP53*, *NOTCH1*, *PIK3CA*; miR-21 dysregulation	Supports precision medicine, miRNA biomarker discovery, and targeted therapies	[70,71]
Dongre et al.	Custom NGS panel in FFPE HNSCC samples	Reliable mutation detection in samples preserved up to 17 years; *TP53*, *FAT1*, *FLG* (HPV−), *FGFR3* (HPV+); mutations correlated with poor differentiation and survival	Enables retrospective analysis, correlates mutation burden with outcomes, confirms TCGA data	[20]
Taiwanese cohort study	Differentiation between recurrence and second primary tumors	NGS identified unique mutations in *SYNE1*, *TP53*, *CDKN2A*; reclassified misdiagnosed recurrences	Improves diagnostic accuracy, guides individualized treatment, refines classification	[21]

**Table 9:** Optical imaging technologies in the diagnosis and management of HNSCC and OSCC.

Study/Authors	Technology	Key Findings	Clinical Implications	References
General review on optical imaging in HNSCC	Fluorescence, NBI, Raman, OCT, HSI, PAI	Imaging techniques offer real-time, non-invasive solutions for early detection and intraoperative guidance	Enhances diagnosis, reduces surgical margin errors, improves outcomes	[72,73]
FMI vs. NBI in OSCC surgery	Fluorescence Molecular Imaging (FMI), Narrow-Band Imaging (NBI)	FMI superior in submucosal extension detection; NBI cost-effective for early-stage lesions	Improves tumor margin delineation and surgical planning	[74]
SI-FLIM in oral dysplasia	SI-FLIM	Enhanced depth-resolved NADH fluorescence; better differentiation of mild dysplasia from normal tissue	Promising for early OSCC detection with higher diagnostic accuracy	[22]
Romano et al.	Toluidine blue, Lugol’s iodine, Autofluorescence, NBI, OCT, Ultrasound, Confocal Microscopy	Proposes 3-step diagnostic model integrating multiple non-invasive tools	Reduces biopsy reliance, improves early diagnosis and patient compliance	[75]

**Table 10:** Therapeutic strategies and oncologic outcomes in OSCC.

Therapeutic Approach	Key Findings	Clinical Implications	References
Surgical Resection	Margin width should be adjusted by tumor stage; narrower margins may suffice in early-stage OSCC; wider margins are necessary in advanced stages due to microscopic spread.	Personalized margin criteria may reduce recurrence without excessive tissue sacrifice.	[76]
CCRT vs. PORT	CCRT improved survival in pT2N1 OSCC compared to PORT; no benefit seen in pT1 cases.	CCRT may be preferred for selected advanced-stage OSCC, while PORT suffices in early-stage disease.	[77]
CCRT (cisplatin + docetaxel)	Safe and effective with response rate comparable to standard regimens; lower incidence of high-grade toxicity.	A feasible alternative to conventional CCRT in advanced OSCC.	[23]
Refusal of AT	AT refusal associated with 34% higher recurrence and poorer recurrence-free and overall survival.	Highlights importance of AT in advanced OSCC; aids clinician counseling.	[78]
Adjuvant Radiotherapy	Indicated in cases with positive margins or nodal metastasis.	Essential for locoregional control in high-risk patients.	[79]
NACT	May reduce tumor size and margin positivity, but lacks survival benefit.	Considered for facilitating conservative surgery; not a survival-enhancing intervention.	[80,81,82]
Neoadjuvant Immunotherapy (ICIs)	Promising results in shrinking tumors and converting inoperable cases to respectable.	Represents a novel preoperative strategy in selected cases.	[24]
R/M HNSCC Treatment	Limited survival gains; OMCT offers a low-toxicity, cost-effective option.	Suitable for frail patients; emphasizes QOL over aggressive regimens.	[83,84]
Personalized Chemotherapy/Multi-omics	Enables molecular stratification and therapeutic tailoring.	Advances precision oncology in HNSCC.	[25]
CRT-related Toxicities	Physiology: mucositis, dysphagia, nausea; Psychology: depression, anxiety.	Necessitates holistic management and supportive care integration.	[85]

## Data Availability

Not applicable.

## Abbreviations

The following abbreviations are used in this manuscript:

OSCC	Oral Squamous Cell Carcinoma
GLOBOCAN	Global Cancer Observatory
HNSCC	Head and Neck Squamous Cell Carcinoma
OPMDs	Oral Potentially Malignant Disorders
EVs	Extracellular Vesicles
WMS	Whole Mouth Saliva
miRNAs	MicroRNAs
NGS	Next-Generation Sequencing
TP53	Tumor Protein p53
CDKN2A	Cyclin Dependent Kinase Inhibitor 2A
EGFR	Epidermal Growth Factor Receptor
PIK3CA	Phosphatidylinositol-4,5-Bisphosphate 3-Kinase Catalytic Subunit Alpha
NOTCH1	Neurogenic locus notch homolog protein 1
FFPE	Formalin-Fixed Paraffin-Embedded
SPTs	Second Primary Tumors
TME	Tumor Microenvironment
PD-L1	Programmed Death-Ligand 1
qRT-PCR	Quantitative Real-Time Polymerase Chain Reaction
FMI	Fluorescence Molecular Imaging
NBI	Narrow Band Imaging
OCT	Optical Coherence Tomography
SI-FLIM	Structured Illumination Fluorescence Lifetime Imaging Microscopy
FLIM	Fluorescence Lifetime Imaging Microscopy
US	Ultrasound
CM	Confocal Microscopy
CCRT	Concurrent Chemoradiotherapy
PORT	Postoperative Radiotherapy
AT	Adjuvant Therapy
CRT	Chemoradiotherapy
NACT	Neoadjuvant Chemotherapy
ICIs	Immune Checkpoint Inhibitors
OMCT	Oral Metronomic Chemotherapy
QOL	Quality of Life
TCGA	The Cancer Genome Atlas
AI	Artificial Intelligence
TIME	Tumor Immune Microenvironment
LVI	Lymphovascular Invasion
DHA	Dihydroartemisinin
EMT	Epithelial–Mesenchymal Transition
R/M HNSCC	Recurrent or Metastatic Head and Neck Squamous Cell Carcinoma
OC	Oral Cancer
DEmiRs	Differentially Expressed miRNAs
DEGs	Differentially Expressed Genes
PPI	Protein–protein iInteraction
PNI	Perineural Invasion
ctDNA	Circulating Tumor DNA
cfRNA	Cell-Free RNA
PER1	Directly Targeting Period 1
PON2	Paraoxonase-2
PD-1	Programmed Death-1

## References

[1] Tranby E.P., Heaton L.J., Tomar S.L., Kelly A.L., Fager G.L., Backley M., Frantsve-Hawley J. (2022). Oral Cancer Prevalence, Mortality, and Costs in Medicaid and Commercial Insurance Claims Data. Cancer Epidemiol. Biomark. Prev..

[2] Johnson D.E., Burtness B., Leemans C.R., Lui V.W.Y., Bauman J.E., Grandis J.R. (2020). Head and neck squamous cell carcinoma. Nat. Rev. Dis. Primers.

[3] Dong L., Xue L., Cheng W., Tang J., Ran J., Li Y. (2024). Comprehensive survival analysis of oral squamous cell carcinoma patients undergoing initial radical surgery. BMC Oral. Health.

[4] Ravikumar L., Velmurugan R. (2024). Innovations in early detection of oral cancer: Advancing diagnostic technologies and reducing global disparities. Oral. Oncol. Rep..

[5] Nagdeve S.N., Suganthan B., Ramasamy R.P. (2025). Perspectives on the Application of Biosensors for the Early Detection of Oral Cancer. Sensors.

[6] Hashemi M., Khoushab S., Aghmiuni M.H., Anaraki S.N., Alimohammadi M., Taheriazam A., Farahani N., Entezari M. (2024). Non-coding RNAs in oral cancer: Emerging biomarkers and therapeutic frontier. Heliyon.

[7] Ravindran S., Ranganathan S., Karthikeyan R., Nandini J., Shanmugarathinam A., Kannan S.K., Prasad K.D., Marri J., Rajaganapathi K. (2025). The role of molecular biomarkers in the diagnosis, prognosis, and treatment stratification of oral squamous cell carcinoma: A comprehensive review. J. Liq. Biopsy.

[8] Pekarek L., Garrido-Gil M.J., Sanchez-Cendra A., Cassinello J., Pekarek T., Fraile-Martinez O., Garcia-Montero C., Lopez-Gonzalez L., Rios-Parra A., Alvarez-Mon M. (2023). Emerging histological and serological biomarkers in oral squamous cell carcinoma: Applications in diagnosis, prognosis evaluation and personalized therapeutics (Review). Oncol. Rep..

[9] Arabi K., Nazemi Salman B., Rahimzadeh-Bajgiran F., Moghbeli M., Moghadas S., Saburi E. (2025). miRNAs in oral cancer; diagnostic and prognostic roles. Gene.

[10] Park Y.N., Ryu J.K., Ju Y. (2024). The Potential MicroRNA Diagnostic Biomarkers in Oral Squamous Cell Carcinoma of the Tongue. Curr. Issues Mol. Biol..

[11] Liu Y., Yang Z., Pu J.J., Zhong J., Khoo U.S., Su Y.X., Zhang G. (2025). Proteogenomic characterisation of primary oral cancer unveils extracellular matrix remodelling and immunosuppressive microenvironment linked to lymph node metastasis. Clin. Transl. Med..

[12] Resurreccion E.P., Fong K.W. (2022). The Integration of Metabolomics with Other Omics: Insights into Understanding Prostate Cancer. Metabolites.

[13] Kinane D.F., Gabert J., Xynopoulos G., Guzeldemir-Akcakanat E. (2024). Strategic approaches in oral squamous cell carcinoma diagnostics using liquid biopsy. Periodontol 2000.

[14] Vinay V., Jodalli P., Chavan M.S., Buddhikot C.S., Luke A.M., Ingafou M.S.H., Reda R., Pawar A.M., Testarelli L. (2025). Artificial Intelligence in Oral Cancer: A Comprehensive Scoping Review of Diagnostic and Prognostic Applications. Diagnostics.

[15] Prasad M., Sekar R., Priya M.D.L., Varma S.R., Karobari M.I. (2024). A new perspective on diagnostic strategies concerning the potential of saliva-based miRNA signatures in oral cancer. Diagn. Pathol..

[16] Balakittnen J., Ekanayake Weeramange C., Wallace D.F., Duijf P.H.G., Cristino A.S., Hartel G., Barrero R.A., Taheri T., Kenny L., Vasani S. (2024). A novel saliva-based miRNA profile to diagnose and predict oral cancer. Int. J. Oral. Sci..

[17] Patel A., Patel S., Patel P., Mandlik D., Patel K., Tanavde V. (2022). Salivary Exosomal miRNA-1307-5p Predicts Disease Aggressiveness and Poor Prognosis in Oral Squamous Cell Carcinoma Patients. Int. J. Mol. Sci..

[18] He L., Ping F., Fan Z., Zhang C., Deng M., Cheng B., Xia J. (2020). Salivary exosomal miR-24-3p serves as a potential detective biomarker for oral squamous cell carcinoma screening. Biomed. Pharmacother..

[19] Bozyk N., Tang K.D., Zhang X., Batstone M., Kenny L., Vasani S., Punyadeera C. (2023). Salivary exosomes as biomarkers for early diagnosis of oral squamous cell carcinoma. Oral. Oncol. Rep..

[20] Dongre H.N., Haave H., Fromreide S., Erland F.A., Moe S.E.E., Dhayalan S.M., Riis R.K., Sapkota D., Costea D.E., Aarstad H.J. (2021). Targeted Next-Generation Sequencing of Cancer-Related Genes in a Norwegian Patient Cohort With Head and Neck Squamous Cell Carcinoma Reveals Novel Actionable Mutations and Correlations With Pathological Parameters. Front. Oncol..

[21] Liu T.Y., Lee C.C., Chen Y.C., Chang Y.S., Huang H.Y., Lee Y.T., Yen J.C., Chao D., Chang J.G. (2022). Mutation Analysis of Second Primary Tumors in Oral Cancer in Taiwanese Patients through Next-Generation Sequencing. Diagnostics.

[22] Hinsdale T.A., Malik B.H., Cheng S., Benavides O.R., Giger M.L., Wright J.M., Patel P.B., Jo J.A., Maitland K.C. (2021). Enhanced detection of oral dysplasia by structured illumination fluorescence lifetime imaging microscopy. Sci. Rep..

[23] Sato K., Hayashi Y., Watanabe K., Yoshimi R., Hibi H. (2019). Concurrent chemoradiotherapy with intravenous cisplatin and docetaxel for advanced oral cancer. Nagoya J. Med. Sci..

[24] Vishwani A., Varghese B.T., Thomas S., Kumar A., Kaur J., Sharma A. (2024). Neoadjuvant immunotherapy in advanced oral cancer: Emerging treatment paradigms. Oral. Oncol. Rep..

[25] Liu X.H., Wang G.R., Zhong N.N., Wang W.Y., Liu B., Li Z., Bu L.L. (2025). Multi-omics in immunotherapy research for HNSCC: Present situation and future perspectives. NPJ Precis. Oncol..

[26] Adorno-Farias D., Morales-Pison S., Gischkow-Rucatti G., Margarit S., Fernandez-Ramires R. (2024). Genetic and epigenetic landscape of early-onset oral squamous cell carcinoma: Insights of genomic underserved and underrepresented populations. Genet. Mol. Biol..

[27] Jiang X., Ye J., Dong Z., Hu S., Xiao M. (2019). Novel genetic alterations and their impact on target therapy response in head and neck squamous cell carcinoma. Cancer Manag. Res..

[28] Zhou G., Liu Z., Myers J.N. (2016). TP53 Mutations in Head and Neck Squamous Cell Carcinoma and Their Impact on Disease Progression and Treatment Response. J. Cell Biochem..

[29] Raghavi S., Anbarasu K. (2024). Unravelling the role of key genes in oral cancer progression: A comprehensive review. Oral. Oncol. Rep..

[30] Lakshmipriya T., Gopinath S.C.B. (2024). Monitoring changes in the P53 gene mutation to diagnose oral cancer. Oral. Oncol. Rep..

[31] Hyodo T., Kuribayashi N., Fukumoto C., Komiyama Y., Shiraishi R., Kamimura R., Sawatani Y., Yaguchi E., Hasegawa T., Izumi S. (2022). The mutational spectrum in whole exon of p53 in oral squamous cell carcinoma and its clinical implications. Sci. Rep..

[32] Shi Y., Xie T., Wang B., Wang R., Cai Y., Yuan B., Gleber-Netto F.O., Tian X., Rodriguez-Rosario A.E., Osman A.A. (2022). Mutant p53 drives an immune cold tumor immune microenvironment in oral squamous cell carcinoma. Commun. Biol..

[33] Giri R., Hota S.K., Senapati U. (2023). Expression of TP53 in oral squamous cell carcinoma and its correlation with adverse histopathological features. J. Cancer Res. Ther..

[34] Lin T.Y., Liu K.Y.P., Novack R., Mattu P.S., Ng T.L., Hoang L.N., Prisman E., Poh C.F., Ko Y.C.K. (2024). Abnormal p53 Immunohistochemical Patterns Are Associated with Regional Lymph Node Metastasis in Oral Cavity Squamous Cell Carcinoma at Time of Surgery. Mod. Pathol..

[35] Jefferies S., Edwards S.M., Hamoudi R.A., A’Hern R., Foulkes W., Goldgar D., Eeles R., Collaborators M.P.T. (2001). No germline mutations in CDKN2A (p16) in patients with squamous cell cancer of the head and neck and second primary tumours. Br. J. Cancer.

[36] Perez-Sayans M., Suarez-Penaranda J.M., Gayoso-Diz P., Barros-Angueira F., Gandara-Rey J.M., Garcia-Garcia A. (2011). p16(INK4a)/CDKN2 expression and its relationship with oral squamous cell carcinoma is our current knowledge enough?. Cancer Lett..

[37] Jeong A.R., Forbes K., Orosco R.K., Cohen E.E.W. (2022). Hereditary oral squamous cell carcinoma associated with CDKN2A germline mutation: A case report. J. Otolaryngol. Head. Neck Surg..

[38] Shahnavaz S.A., Bradley G., Regezi J.A., Thakker N., Gao L., Hogg D., Jordan R.C. (2001). Patterns of CDKN2A gene loss in sequential oral epithelial dysplasias and carcinomas. Cancer Res..

[39] Ribeiro F.A., Noguti J., Oshima C.T., Ribeiro D.A. (2014). Effective targeting of the epidermal growth factor receptor (EGFR) for treating oral cancer: A promising approach. Anticancer. Res..

[40] Chen I.H., Chang J.T., Liao C.T., Wang H.M., Hsieh L.L., Cheng A.J. (2003). Prognostic significance of EGFR and Her-2 in oral cavity cancer in betel quid prevalent area cancer prognosis. Br. J. Cancer.

[41] Civico-Ortega J.L., Gonzalez-Ruiz I., Ramos-Garcia P., Cruz-Granados D., Samayoa-Descamps V., Gonzalez-Moles M.A. (2023). Prognostic and Clinicopathological Significance of Epidermal Growth Factor Receptor (EGFR) Expression in Oral Squamous Cell Carcinoma: Systematic Review and Meta-Analysis. Int. J. Mol. Sci..

[42] Laimer K., Spizzo G., Gastl G., Obrist P., Brunhuber T., Fong D., Barbieri V., Jank S., Doppler W., Rasse M. (2007). High EGFR expression predicts poor prognosis in patients with squamous cell carcinoma of the oral cavity and oropharynx: A TMA-based immunohistochemical analysis. Oral. Oncol..

[43] Huang S.F., Chien H.T., Chuang W.Y., Lai C.H., Cheng S.D., Liao C.T., Wang H.M. (2017). Epidermal growth factor receptor intron-1 CA repeat polymorphism on protein expression and clinical outcome in Taiwanese oral squamous cell carcinoma. Sci. Rep..

[44] Rehmani H.S., Issaeva N. (2020). EGFR in head and neck squamous cell carcinoma: Exploring possibilities of novel drug combinations. Ann. Transl. Med..

[45] Wang J., Lv N., Lu X., Yuan R., Chen Z., Yu J. (2021). Diagnostic and therapeutic role of microRNAs in oral cancer (Review). Oncol. Rep..

[46] Ghafouri-Fard S., Gholipour M., Taheri M., Shirvani Farsani Z. (2020). MicroRNA profile in the squamous cell carcinoma: Prognostic and diagnostic roles. Heliyon.

[47] Rajan C., Roshan V.G.D., Khan I., Manasa V.G., Himal I., Kattoor J., Thomas S., Kondaiah P., Kannan S. (2021). MiRNA expression profiling and emergence of new prognostic signature for oral squamous cell carcinoma. Sci. Rep..

[48] Wang X., Zhang S., Wang S., Cao T., Fan H. (2024). Decoding oral cancer: Insights from miRNA expression profiles and their regulatory targets. Front. Mol. Biosci..

[49] Dioguardi M., Spirito F., Iacovelli G., Sovereto D., Laneve E., Laino L., Caloro G.A., Nabi A.Q., Ballini A., Lo Muzio L. (2023). The Potential microRNA Prognostic Signature in HNSCCs: A Systematic Review. Noncoding RNA.

[50] Jakob M., Mattes L.M., Kuffer S., Unger K., Hess J., Bertlich M., Haubner F., Ihler F., Canis M., Weiss B.G. (2019). MicroRNA expression patterns in oral squamous cell carcinoma: Hsa-mir-99b-3p and hsa-mir-100-5p as novel prognostic markers for oral cancer. Head. Neck.

[51] Lu Z., He Q., Liang J., Li W., Su Q., Chen Z., Wan Q., Zhou X., Cao L., Sun J. (2019). miR-31-5p Is a Potential Circulating Biomarker and Therapeutic Target for Oral Cancer. Mol. Ther. Nucleic Acids.

[52] Yu E.H., Tu H.F., Wu C.H., Yang C.C., Chang K.W. (2017). MicroRNA-21 promotes perineural invasion and impacts survival in patients with oral carcinoma. J. Chin. Med. Assoc..

[53] Burtyn O., Borikun T., Rossylna O., Kopchak A., Kravets O. (2024). Clinical Significance of Salivary Mir-21, -155, and -375 in Patients with Squamous Cell Carcinoma of Oral Cavity. Exp. Oncol..

[54] Patel A., Patel S., Patel P., Mandlik D., Patel K., Tanavde V. (2022). Salivary exosomal miR-1307-5p predicts disease aggressiveness and poor prognosis in oral squamous cell carcinoma patients. bioRxiv.

[55] Yang R., Li T., Zhang S., Shui C., Ma H., Li C. (2024). The effect of circulating tumor DNA on the prognosis of patients with head and neck squamous cell carcinoma: A systematic review and meta-analysis. BMC Cancer.

[56] Xu Q., Li X. (2024). Tumor-derived extracellular vesicles in the immune microenvironment of head and neck squamous cell carcinoma: Foe or future?. J. Stomatol. Oral. Maxillofac. Surg..

[57] Cristaldi M., Mauceri R., Di Fede O., Giuliana G., Campisi G., Panzarella V. (2019). Salivary Biomarkers for Oral Squamous Cell Carcinoma Diagnosis and Follow-Up: Current Status and Perspectives. Front. Physiol..

[58] AlAli A.M., Walsh T., Maranzano M. (2020). CYFRA 21-1 and MMP-9 as salivary biomarkers for the detection of oral squamous cell carcinoma: A systematic review of diagnostic test accuracy. Int. J. Oral. Maxillofac. Surg..

[59] Vats R., Yadav P., Bano A., Wadhwa S., Bhardwaj R. (2024). Salivary biomarkers in non-invasive oral cancer diagnostics: A comprehensive review. J. Appl. Oral. Sci..

[60] Bastias D., Maturana A., Marin C., Martinez R., Niklander S.E. (2024). Salivary Biomarkers for Oral Cancer Detection: An Exploratory Systematic Review. Int. J. Mol. Sci..

[61] Senevirathna K., Mahakapuge T.A.N., Jayawardana N.U., Rajapakse J., Gamage C.U., Seneviratne B., Perera U., Kanmodi K.K., Jayasinghe R.D. (2024). Diagnostic potential of salivary IL-1beta, IL-8, SAT, S100P, and OAZ1 in oral squamous cell carcinoma, oral submucous fibrosis, and oral lichen planus based on findings from a Sri Lankan cohort. Sci. Rep..

[62] Hu Y., Xu M., Liu M., Peng H. (2025). Comparison of saliva and blood derived cell free RNAs for detecting oral squamous cell carcinoma. Sci. Rep..

[63] Ghiyasimoghaddam N., Shayan N., Mirkatuli H.A., Baghbani M., Ameli N., Ashari Z., Mohtasham N. (2024). Does circulating tumor DNA apply as a reliable biomarker for the diagnosis and prognosis of head and neck squamous cell carcinoma?. Discov. Oncol..

[64] Sanesi L., Mori G., Troiano G., Ballini A., Valzano F., Dioguardi M., Muzio L.L., Magalhaes M., Caponio V.C.A. (2024). Salivary exosomal microRNA profile as biomonitoring tool for diagnosis and prognosis of patients with head and neck squamous cell carcinoma: A systematic review. Arch. Oral. Biol..

[65] Maheswari T.N.U., Venugopal A., Sureshbabu N.M., Ramani P. (2018). Salivary micro RNA as a potential biomarker in oral potentially malignant disorders: A systematic review. Tzu Chi Med. J..

[66] Gai C., Camussi F., Broccoletti R., Gambino A., Cabras M., Molinaro L., Carossa S., Camussi G., Arduino P.G. (2018). Salivary extracellular vesicle-associated miRNAs as potential biomarkers in oral squamous cell carcinoma. BMC Cancer.

[67] Zhang Y., Liu J., Liu S., Yu L., Liu S., Li M., Jin F. (2023). Extracellular vesicles in oral squamous cell carcinoma: Current progress and future prospect. Front. Bioeng. Biotechnol..

[68] Wang Q., Sun J., Jiang H., Yu M. (2025). Emerging roles of extracellular vesicles in oral and maxillofacial areas. Int. J. Oral. Sci..

[69] Li R., Zhou Y., Zhang M., Xie R., Duan N., Liu H., Qin Y., Ma J., Li Z., Ye P. (2023). Oral squamous cell carcinoma-derived EVs promote tumor progression by regulating inflammatory cytokines and the IL-17A-induced signaling pathway. Int. Immunopharmacol..

[70] Sasahira T., Kurihara-Shimomura M., Shimojjukoku Y., Shima K., Kirita T. (2022). Searching for New Molecular Targets for Oral Squamous Cell Carcinoma with a View to Clinical Implementation of Precision Medicine. J. Pers. Med..

[71] Kim S., Lee J.W., Park Y.S. (2020). The Application of Next-Generation Sequencing to Define Factors Related to Oral Cancer and Discover Novel Biomarkers. Life.

[72] van Schaik J.E., Halmos G.B., Witjes M.J.H., Plaat B.E.C. (2021). An overview of the current clinical status of optical imaging in head and neck cancer with a focus on Narrow Band imaging and fluorescence optical imaging. Oral. Oncol..

[73] Zhang Y., Li Z., Zhang C., Shao C., Duan Y., Zheng G., Cai Y., Ge M., Xu J. (2025). Recent advances of photodiagnosis and treatment for head and neck squamous cell carcinoma. Neoplasia.

[74] de Wit J.G., van Schaik J.E., Voskuil F.J., Vonk J., de Visscher S., Schepman K.P., van der Laan B., Doff J.J., van der Vegt B., Plaat B.E.C. (2022). Comparison of narrow band and fluorescence molecular imaging to improve intraoperative tumour margin assessment in oral cancer surgery. Oral. Oncol..

[75] Romano A., Di Stasio D., Petruzzi M., Fiori F., Lajolo C., Santarelli A., Lucchese A., Serpico R., Contaldo M. (2021). Noninvasive Imaging Methods to Improve the Diagnosis of Oral Carcinoma and Its Precursors: State of the Art and Proposal of a Three-Step Diagnostic Process. Cancers.

[76] Jang J.Y., Choi N., Jeong H.S. (2022). Surgical Extent for Oral Cancer: Emphasis on a Cut-Off Value for the Resection Margin Status: A Narrative Literature Review. Cancers.

[77] Chang C.C., Wu Y.T., Lu H.H., Cheng Y.J., Tsai M.H. (2025). The role of postoperative radiotherapy or chemoradiation in pT1-2N1M0 oral squamous cell carcinoma. J. Formos. Med. Assoc..

[78] Mrosk F., Doll C., Scheer J., Neumann F., Hofmann E., Kreutzer K., Voss J., Rubarth K., Beck M., Heiland M. (2023). Oncologic Outcome in Advanced Oral Squamous Cell Carcinoma After Refusal of Recommended Adjuvant Therapy. JAMA Otolaryngol. Head. Neck Surg..

[79] Dhawan A., Bonanthaya K., Panneerselvam E., Manuel S., Kumar V.V., Rai A. (2021). Adjunctive Therapy in Oral Cancer. Oral and Maxillofacial Surgery for the Clinician.

[80] Geiger J.L., Adelstein D.J. (2020). Chemotherapy in the definitive management of oral cancers: Where do we stand today?. Oral. Oncol..

[81] Malik A., Vishnoi K., Noronha V., Prabhash K. (2025). A nuanced review of neoadjuvant therapies in oral cancer. Expert. Rev. Anticancer. Ther..

[82] Kende P., Mathur Y., Varte V., Tayal S., Patyal N., Landge J. (2024). The efficacy of neoadjuvant chemotherapy as compared to upfront surgery for the management of oral squamous cell carcinoma: A systematic review and meta-analysis. Int. J. Oral. Maxillofac. Surg..

[83] Rajendra A., Noronha V., Joshi A., Patil V.M., Menon N., Prabhash K. (2020). Palliative chemotherapy in head and neck cancer: Balancing between beneficial and adverse effects. Expert. Rev. Anticancer. Ther..

[84] Kumar N.A.N., Dikhit P.S., Jose A., Mehta V., Pai A., Kudva A., Rao M. (2024). Oral Metronomic Chemotherapy in Advanced and Metastatic Oral Squamous Cell Carcinoma: A Need of the Hour. J. Maxillofac. Oral. Surg..

[85] Bhutani R., Singh R., Mishra A., Baluni P. (2024). The adverse impact of chemo-radiotherapy on the quality of life of oral cancer patients: A review. Oral. Oncol. Rep..

[86] Warnakulasuriya S. (2009). Global epidemiology of oral and oropharyngeal cancer. Oral. Oncol..

[87] Lingen M.W., Kalmar J.R., Karrison T., Speight P.M. (2008). Critical evaluation of diagnostic aids for the detection of oral cancer. Oral. Oncol..

[88] Leemans C.R., Snijders P.J.F., Brakenhoff R.H. (2018). The molecular landscape of head and neck cancer. Nat. Rev. Cancer.

[89] Melo-Alvim C., Neves M.E., Santos J.L., Abrunhosa-Branquinho A.N., Barroso T., Costa L., Ribeiro L. (2022). Radiotherapy, Chemotherapy and Immunotherapy-Current Practice and Future Perspectives for Recurrent/Metastatic Oral Cavity Squamous Cell Carcinoma. Diagnostics.

[90] Menditti D., Santagata M., Imola G., Staglianò S., Vitagliano R., Boschetti C.E., Inchingolo A.M. (2023). Personalized Medicine in Oral Oncology: Imaging Methods and Biological Markers to Support Diagnosis of Oral Squamous Cell Carcinoma (OSCC): A Narrative Literature Review. J. Pers. Med..

[91] Muralidharan S., Nikalje M., Subramaniam T., Koshy J.A., Koshy A.V., Bangera D. (2025). A Narrative Review on Oral Squamous Cell Carcinoma. J. Pharm. Bioallied Sci..

[92] Campagna R., Pozzi V., Salvucci A., Togni L., Mascitti M., Sartini D., Salvolini E., Santarelli A., Lo Muzio L., Emanuelli M. (2023). Paraoxonase-2 expression in oral squamous cell carcinoma. Hum. Cell.

[93] Parween F., Gupta R.D. (2021). Insights into the role of paraoxonase 2 in human pathophysiology. J. Biosci..

[94] Kamal M.V., Prabhu K., Sharan K., Pai A., Chakrabarty S., Damerla R.R., Shetty P.S., Belle V.S., Rao M., Kumar N.A.N. (2025). Investigation of the Molecular Mechanisms of Paraoxonase-2 Mediated Radiotherapy and Chemotherapy Resistance in Oral Squamous Cell Carcinoma. Clin. Transl. Sci..

[95] Ashwini R., Narayan M., Rajkumar K. (2024). Diagnostic and prognostic markers of oral squamous cell carcinoma- a detailed review. Oral. Oncol. Rep..

[96] Mahajan A., Mohanty S., Ghosh S., Urs A.B., Khurana N., Gupta S. (2017). Sarcomatoid Carcinoma of the Oral Cavity: A Diagnostic Dilemma. Case Rep. Dent..

[97] Harnischfeger N., Szabo L., Kretzschmar K. (2025). Establishment and Characterization of Patient-Derived Oral Cancer Organoids.

[98] Adeola H.A., Bello I.O., Aruleba R.T., Francisco N.M., Adekiya T.A., Adefuye A.O., Ikwegbue P.C., Musaigwa F. (2022). The Practicality of the Use of Liquid Biopsy in Early Diagnosis and Treatment Monitoring of Oral Cancer in Resource-Limited Settings. Cancers.

